# Drug delivery by sonosensitive liposome and microbubble with acoustic-lens attached ultrasound: an in vivo feasibility study in a murine melanoma model

**DOI:** 10.1038/s41598-023-42786-8

**Published:** 2023-09-22

**Authors:** Jun Hong Park, Byung Chul Lee, Young Chan Seo, Jung Hoon Kim, Da Jung Kim, Hak Jong Lee, Hyungwon Moon, Seunghyun Lee

**Affiliations:** 1 Bionics Research Center, Korea Institute of Science and Technology (KIST), Seoul, 02792 Republic of Korea; 2 Division of Bio-Medical Science & Technology, KIST School, University of Science & Technology (UST), Seoul, 02792 Republic of Korea; 3 KHU-KIST Department of Converging Science and Technology, Kyung Hee University, Seoul, 02447 Republic of Korea; 4 Department of Medical Device Development, Seould National University College of Medicine, Seoul, 03080 Republic of Korea; 5 Department of Radiology, Seoul National University Hospital, 101 Daehak-Ro, Jongno-Gu, Seoul, 03080 Republic of Korea; 6 Department of Radiology, Seoul National University College of Medicine, 103 Daehak-Ro, Jongno-Gu, Seoul, 03080 Republic of Korea; 7 Institute of Radiation Medicine, Seoul National University Medical Research Center, 103 Daehak-Ro, Jongno-Gu, Seoul, 03080 Republic of Korea; 8 Metabolomics Core Facility, Department of Transdisciplinary Research and Collaboration, Biomedical Research Institute, Seoul National University Hospital, 101 Daehak-Ro, Jongno-Gu, Seoul, 03080 Republic of Korea; 9 Department of Radiology, Seoul National University Bundang Hospital, 82 Gumi-Ro 173, Bundang-Gu, Seongnam, 13620 Republic of Korea; 10 R&D Center, IMGT Co. Ltd., 172, Dolma-Ro, Bundang-Gu, Seongnam, 13605 Republic of Korea; 11 Innovative Medical Technology Research Institute, Seoul National University Hospital, 101 Daehak-Ro, Jongno-Gu, Seoul, 03080 Republic of Korea; 12 Present Address: Department of Radiology, Stanford University, Stanford, CA 94305 USA

**Keywords:** Biomedical engineering, Biomedical materials, Melanoma, Drug delivery, Preclinical research

## Abstract

Conventional chemotherapy methods have adverse off-target effects and low therapeutic efficiencies of drug release in target tumors. In this study, we proposed a combination therapy of doxorubicin (DOX)-loaded ultrasound (US)-sensitive liposomal nanocarriers (IMP301), microbubbles (MBs) under focused US exposure using convex acoustic lens-attached US (LENS) to tumor treatment. The therapeutic effects of each treatment in a murine melanoma model were evaluated using contrast-enhanced US (CEUS) and micro-computed tomography (micro-CT) imaging, bioluminescence and confocal microscopy imaging, and liquid chromatography–mass spectroscopy (LC/MS) analysis. Tumor-bearing mice were randomly assigned to one of the following groups: (1) G1: IMP301 only (n = 9); (2) G2: IMP301 + LENS (n = 9); (3) G3: IMP301 + MB + LENS (n = 9); (4) G4: DOXIL only (n = 9); and (5) G5: IMP301 without DOXIL group as a control group (n = 4). Ten days after tumor injection, tumor-bearing mice were treated according to each treatment strategy on 10th, 12th, and 14th days from the day of tumor injection. The CEUS images of the tumors in the murine melanoma model clearly showed increased echo signal intensity from MBs as resonant US scattering. The relative tumor volume of the G2 and G3 groups on the micro-CT imaging showed inhibited tumor growth than the reference baseline of the G5 group. DOX signals on bioluminescence and confocal microscopy imaging were mainly located at the tumor sites. LC/MS showed prominently higher intratumoral DOX concentration in the G3 group than in other treated groups. Therefore, this study effectively demonstrates the feasibility of the synergistic combination of IMP301, MBs, and LENS-application for tumor-targeted treatment. Thus, this study can enable efficient tumor-targeted treatment by combining therapy such as IMP301 + MBs + LENS-application.

## Introduction

Melanoma is an aggressive skin cancer associated with resistance and a low response rate to conventional chemotherapy, making it challenging to treat^1^. Traditional chemotherapy is ineffective in treating advanced melanoma and causes adverse effects on healthy tissues due to the wide distribution of drugs despite their toxic effects on tumor cells^2^. Therefore, various drug-loaded carriers have been developed as a locally targeting method that can increase therapeutic efficacy by enhancing drug vascular permeability into the tumor tissue to overcome the disadvantages of conventional chemotherapy^3^.

Liposomal formulations of chemotherapeutic agents, drug-loaded carriers, can accumulate inside the tumor with enhanced permeability and retention effects^4^. However, liposomal chemotherapeutic drugs, such as PEGylated liposomal doxorubicin (DOX) HCl (CAELYX™/DOXIL®), are still limited for the effective treatment of cancer due to a low release of the drug, despite advances in its improved bioavailability and the reduction in systemic side effects due to the liposomal shell^5–7^. Therefore, combining local stimulation-responsive liposomes and ultrasound (US) has recently emerged as a noninvasive and convenient method for stimulating drug release^8^.

The enhanced drug delivery strategy involves the administration of liposomal chemotherapeutic agents with or without microbubbles (MBs) and then exposing the tumor tissues under US pressure. A previous study reported that the combination of DOX-loaded MBs and US provided a diagnosis of melanoma and efficiently delivered DOX to primary melanoma tumors in a mouse model^9^. Recent research has focused on inducing MB cavitation/sonoporation and optimizing drug release from liposomes by maximally delivering ultrasonic pressure using a high-intensity focused US (FUS) device while characterizing the primary melanoma^10^. However, the fixed US beam distribution in bulky and complex high-intensity FUS devices might limit their applicability in treating various cancer morphologies^11^. A previous study showed that a diagnostic phased array US transducer coupled to a concave-shaped acoustic lens diverging US beam for widespread US irradiation is sufficient for US pressure-based drug delivery^12,13^.

**Figure 1:**
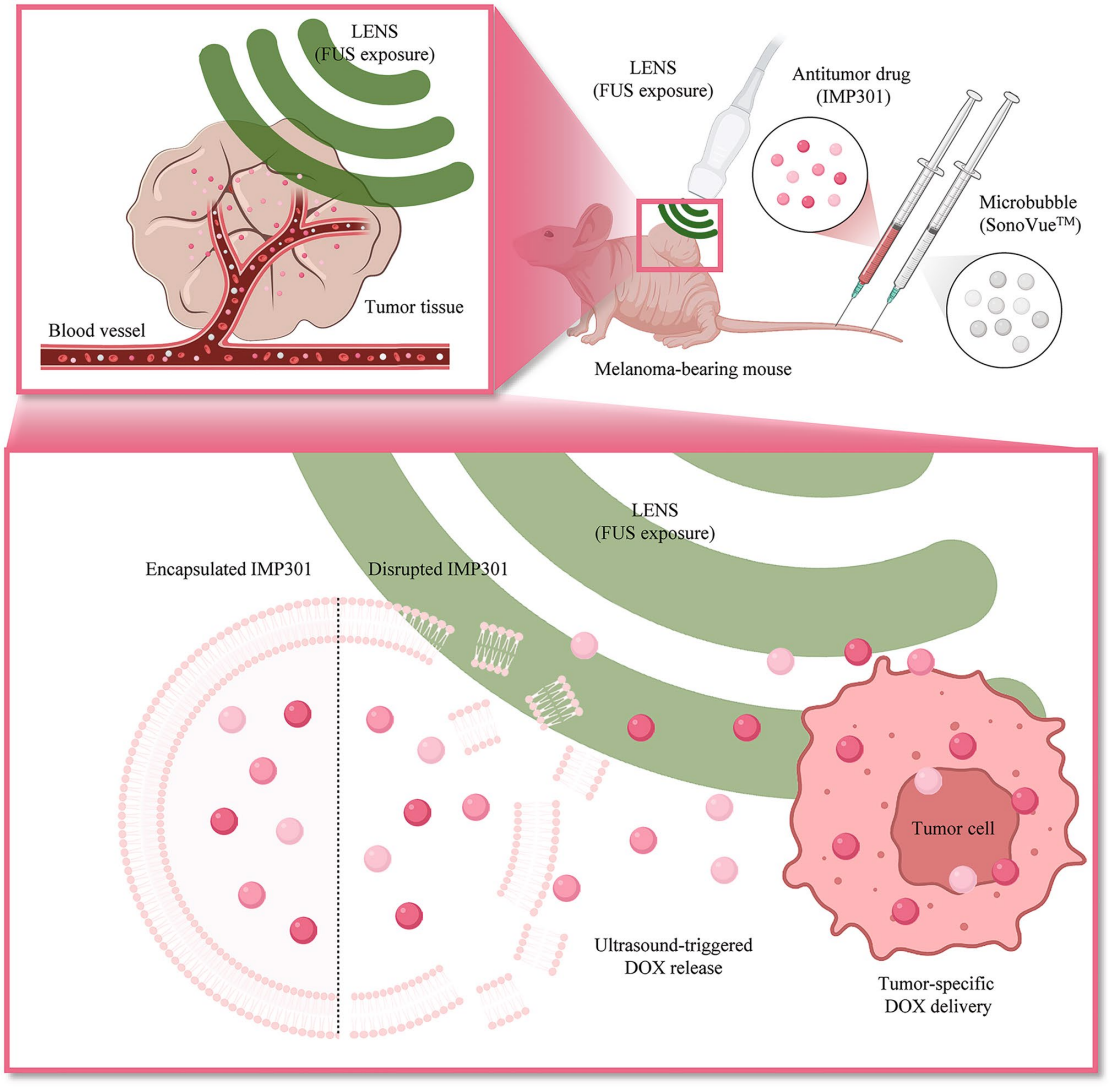
Targeted Drug Delivery System using Acoustic Lens-attached US (LENS) and Sonoresponsive Liposomes. Schematic of the target-specific drug delivery system by acoustic lens-attached US system (LENS) with the antitumor drug-loaded sonoresponsive liposome carrier (IMP301). Upon reaching the target tumor region through the microvasculature, disrupted IMP301 releases an antitumor drug by FUS exposure and MB oscillation.

Herein, we propose a combination of convex acoustic lens-attached US (LENS)-mediated drug delivery system, drug-loaded US-sensitive liposomes, and MBs as a new combination therapy (Fig. 1). This combined therapy (LENS, US-sensitive liposomes, and MBs) enables efficient drug delivery to the tumor.

## Results

### Contrast-Enhanced ultrasound imaging to acoustic lens-attached ultrasound (LENS)-applied effect

**Figure 2:**
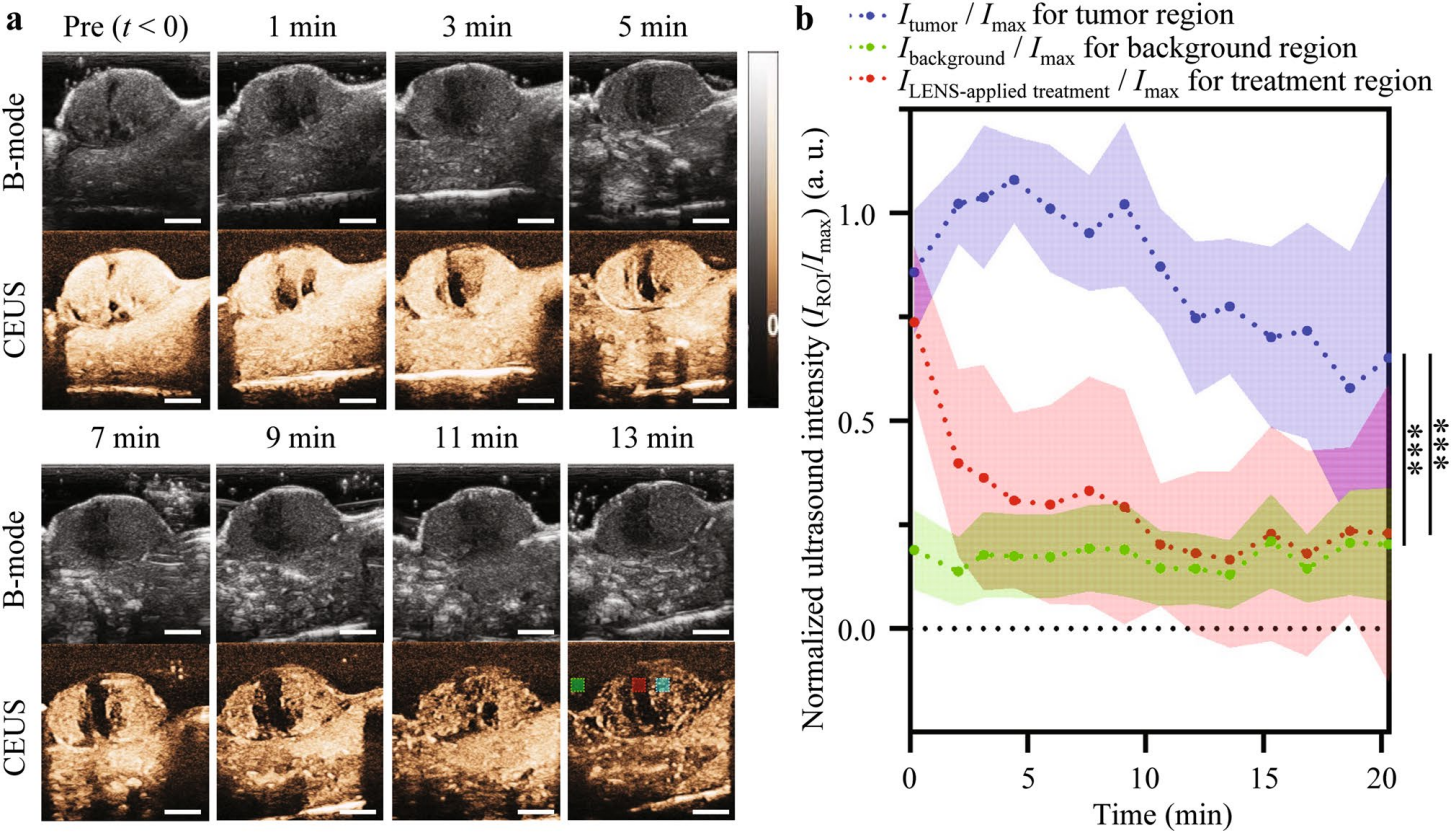
Contrast-Enhanced Ultrasound Imaging of Subcutaneous Tumors under FUS Exposure. (**a**) Time-dependent in vivo representative subcutaneous tumor US images after intravenous injection of the contrast agent (MB) under FUS exposure (Upper panel: B-mode; lower panel: CEUS-mode) (n = 1). Green, red, and cyan dotted boxes in the CEUS image at 13 min indicate region of interests (ROIs) of background, LENS-applied treatment, and tumor areas for the normalized US intensity calculation. (**b**) The normalized US intensity value of CEUS imaging, which is obtained by dividing US intensity in the ROI by the maximum US intensity in the entire CEUS image after injection of the contrast agent over time (*I*_tumor_/*I*_max_ vs. *I*_background_/*I*_max_: ***p < 0.0001; *I*_tumor_/*I*_max_ vs. *I*_LENS-applied treatment_/*I*_max_: ***p < 0.0001). The spot and shaded area represent the mean and standard deviation of the calculated values.

The Contrast-Enhanced US (CEUS) images of the tumors in the murine melanoma model clearly showed increased echo signal intensity from MBs as resonant US scattering (Fig. 2). Although the overall echo signals decreased over time owing to MB dissolution and blood circulation, LENS-applied treatment reduced the echo signals in the targeted region more rapidly (Fig. 2a). Quantitative US signal analysis demonstrated that the normalized US intensity of the treated tumor site sharply decreased immediately after LENS-applied treatment and then converged to a specific value range similar to that of the background after 10 min. However, the normalized US intensity of the untreated tumor sites remains 2.84 times higher than that of the treated tumor site at 20 min after LENS-applied treatment, although it gradually decreased over time (Fig. 2b).

### Therapeutic effect by LENS-applied treatment

The tumor-bearing mice were randomized into one of the following groups: (1) G1: IMP301 only group (*n* = 9); (2) G2: IMP301 with LENS-applied group (*n* = 9); (3) G3: IMP301 with MB injection and LENS-applied group (*n* = 9); (4) G4: DOXIL (*n* = 9); and (5) G5: IMP301 without DOXIL (*n* = 4).

**Figure 3:**
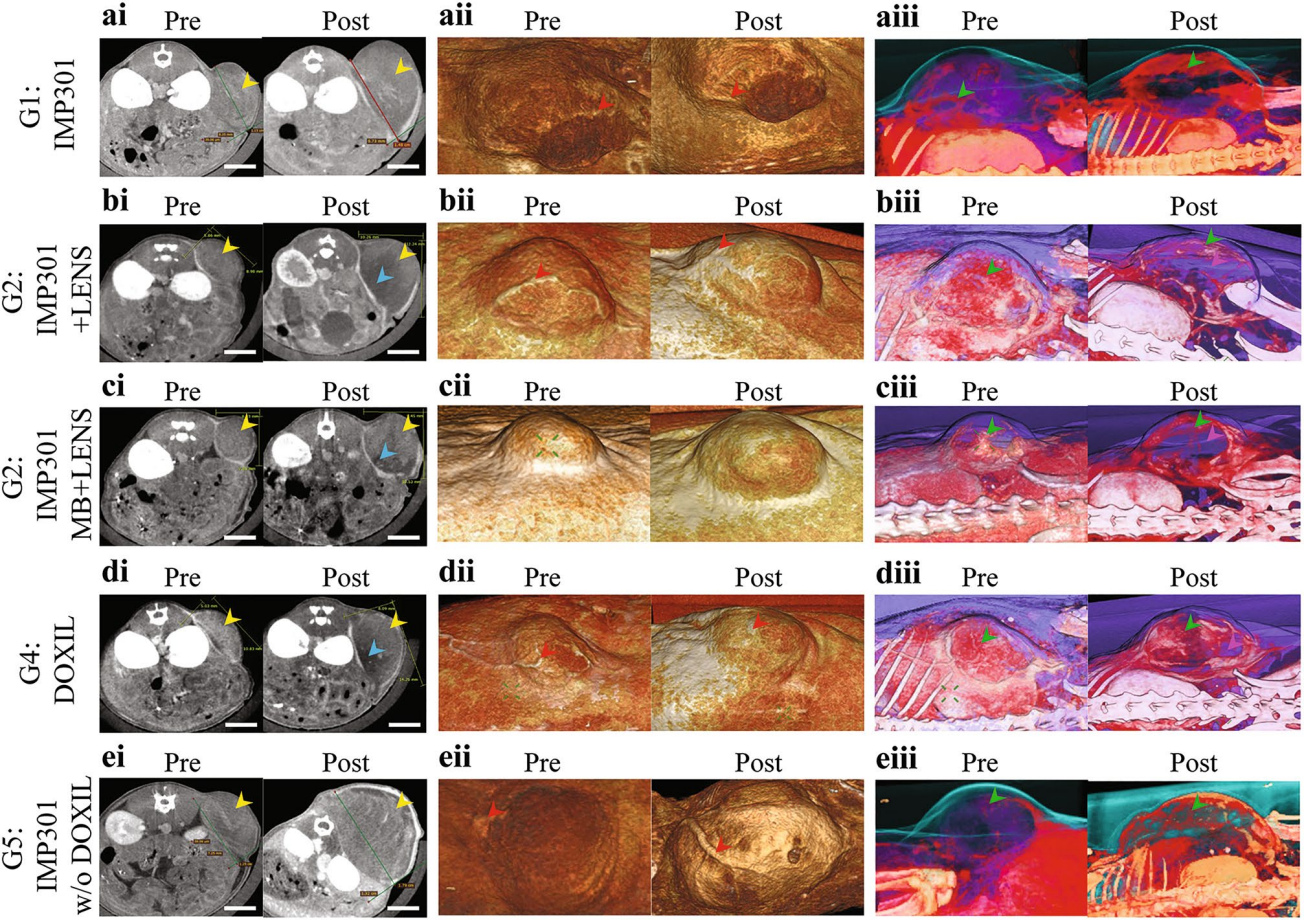
Assessing the Efficacy of the Drug Delivery Treatment in Murine Subcutaneous Melanoma Model: Micro-CT and 3D imaging. (**ai**–**ei**) Micro-CT images, (**aii**–**eii**) 3D skin reconstruction, and (**aiii**–**eiii**) 3D micro-CT reconstruction images of the tumor region in the murine subcutaneous tumor model on the transverse plane before and after each treatment (G1: IMP301, G2: IMP301 + LENS-applied treatment, G3: IMP301 + MB + LENS-applied treatment, G4: DOXIL only and G5: IMP301 without DOXIL). Relative tumor growth for G1, G2, G3, G4 and G5 are 264.6%, 239.6%, 210.4%, 254.2%, and 302.4%, respectively from the micro-CT images. Yellow and blue arrows indicate tumor and tumor necrosis in micro-CT images, respectively. Red arrows indicate extratumoral angiogenesis in 3D skin reconstruction images. Green and magenta arrows indicate intratumoral angiogenesis and intratumoral necrosis in 3D micro-CT reconstruction images. The scale bar indicates 5 mm.

The therapeutic effect of the combination of IMP301, MBs, and LENS-applied treatment (G3) was evaluated by using micro-computed tomography (micro-CT) imaging and 3D reconstruction and compared with those of other treatment groups (Fig. 3a–e). Based on the micro-CT representatives, relative tumor growth was calculated by dividing the tumor volume measured from the micro-CT image before treatment by that after the treatment. The G3 group has a smaller relative tumor growth of 210.4% than the other groups, followed by G2 at 239.6%, G4 at 254.2%, G1 at 264.6%, and G5 at 302.4% (Fig. 3ai–ei). When treating tumors with IMP301 and LENS-applied therapies (G2 and G3 groups), the target-specific therapeutic effect and intratumoral necrosis significantly reduced intratumoral vascular perfusion from tumor angiogenesis, especially at the tumor core sites.

**Figure 4:**
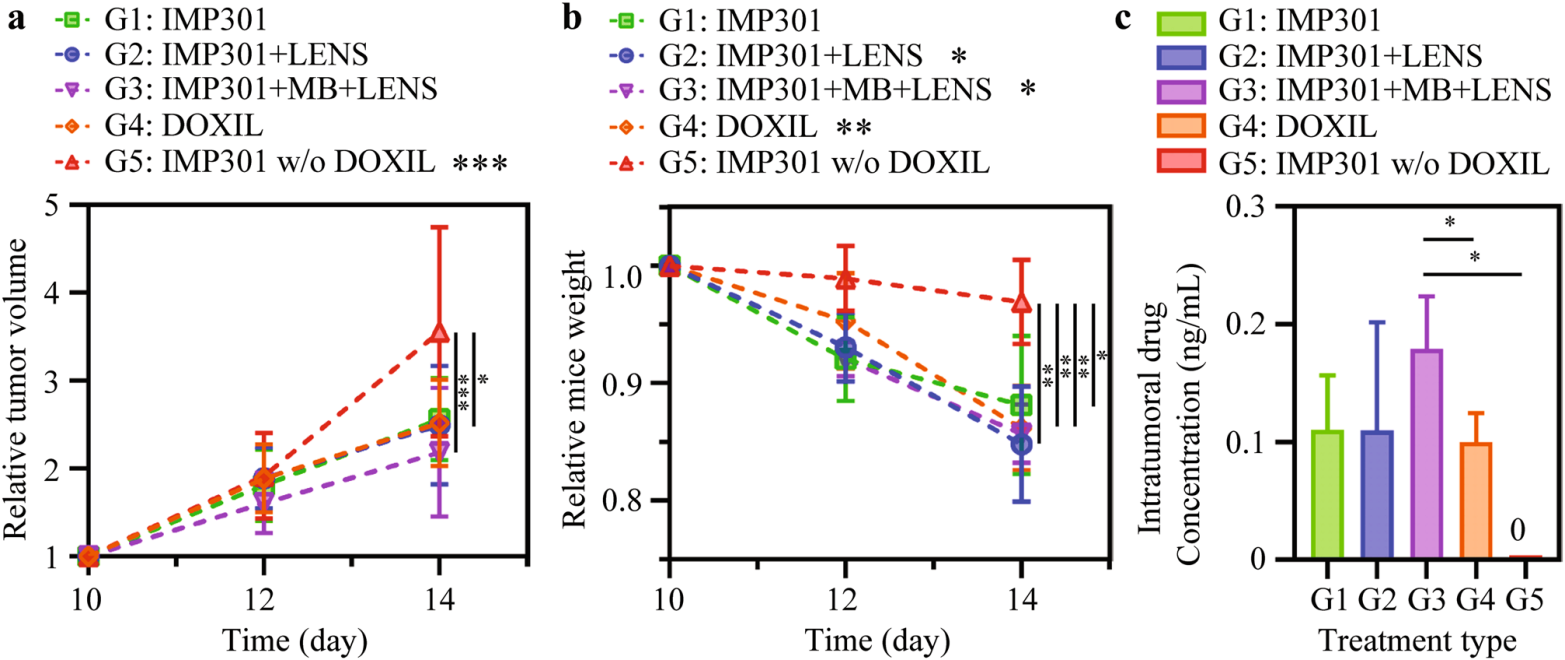
Assessing the Efficacy and Safety of the Drug Delivery Treatment in Murine Subcutaneous Melanoma Model: Relative tumor volume, Relative body weight, and Pharmacokinetic Analysis. (**a**) Relative tumor volume per mouse (G5, 12 days vs. 14 days: ***p < 0.0001; 14 days, G2 vs. G5: *p < 0.05; 14 days, G3 vs. G5: ***p < 0.0001) and (**b**) relative body weight per mouse (G2, 12 days vs. 14 days: *p < 0.05; G3, 12 days vs. 14 days: *p < 0.05; G4, 12 days vs. 14 days: **p < 0.001; 14 days, G1 vs. G5: *p < 0.05; 14 days, G2 vs. G5: **p < 0.001; 14 days, G3 vs. G5: **p < 0.001; 14 days, G4 vs. G5: **p < 0.001). (**c**) DOX concentration within the tumor in the mouse tumor model with each treatment at the end of the study (15 days after the tumor in each treatment) (G3 vs. G4: *p < 0.05; G3 vs. G5: *p < 0.05).

Figure 4a shows relative tumor growth curves with five different treatments, whose relative volumes were defined as absolute volumes divided by absolute volumes on the 10th day after tumor inoculation. The relative tumor volume of the control group, IMP301 without DOXIL (G5), increased dramatically from the 12th day to the 14th day after tumor inoculation (12th day: 1.92 ± 0.49, 14th day: 3.56 ± 1.19) (Table 1). However, tumor growth in DOX involved groups (G1, G2, G3 and G4) slowed (G1, 12th day: 1.81 ± 0.41, 14th day: 2.56 ± 0.46; G2, 12th day: 1.89 ± 0.35, 14th day: 2.49 ± 0.67; G3, 12th day: 1.61 ± 0.34, 14th day: 2.19 ± 0.73; G4, 12th day: 1.89 ± 0.39, 14th day: 2.52 ± 0.49) (Table 1). Upon comparing the relative tumor volume of the IMP301 with additional treatment groups (G2 and G3) to that of G5 on the 14th day, we observed significant differences among them (G2 vs. G5: *p < 0.05; G3 vs. G5: ***p < 0.0001 at 14 days). However, no significant tumor volume difference was detected between G1 and G5 groups. As shown in Fig. 4b, the curves of the relative body weight of mice from each treatment, defined as absolute mouse weight divided by absolute mouse weight on the 10th day after tumor inoculation, were monitored to investigate their therapeutic efficacy against the tumor. The weight of the mice exhibited an inversely proportional trend to the relative tumor growth (Fig. 4b). This demonstrated that the combined effect of IMP301 and LENS-applied treatment significantly reduced the body weight of the mice (G2 and G3 groups) (*p* < 0.01) over time (G2, 12th day: 0.93 ± 0.03, 14th day: 0.85 ± 0.05; G3, 12th day: 0.92 ± 0.01, 14th day: 0.86 ± 0.02), while there were no significant variations in body weight in G1 and G5 groups (G1, 12th day: 0.92 ± 0.04, 14th day: 0.88 ± 0.06; G5, 12th day: 0.99 ± 0.03, 14th day: 0.97 ± 0.04) (Table 1). The intratumoral DOX concentration in the G3 group was prominently higher than that in other IMP301 treated groups (G1 and G2), maintaining considerable DOX concentration in the tumor to improve the efficacy of treatment (G1, 110.43 ± 46.01 ng/mL; G2, 109.93 ± 91.79 ng/mL; G3, 179.22 ± 44.34 ng/mL; G4, 99.82 ± 24.58 ng/mL; G5, 0.00 ± 0.00 ng/mL; Fig. 4c). We observed a statistically significant difference in the intratumoral DOX concentration between the G3 group and both the G4 and G5 groups (G3 vs. G4: *p < 0.05; G3 vs. G5: *p < 0.05). These findings indicate the potential therapeutic effect and the in vivo feasibility of the additional treatments (LENS and MB + LENS) when combined with IMP301.

**Table 1:** Assessing the efficacy and safety of the drug delivery treatment in murine subcutaneous melanoma model.

Group	Relative tumor volume (Relative to 10 days)		Relative mice weight (Relative to 10 days)		Intratumoral drug concentration (ng/mL) (15 days after treatment)
	12 days	14 days	12 days	14 days	
G1: IMP301	1.81 ± 0.41	2.56 ± 0.46	0.92 ± 0.04	0.88 ± 0.06	110.43 ± 46.01
G2: IMP301 + LENS	1.89 ± 0.35	2.49 ± 0.67	0.93 ± 0.03	0.85 ± 0.05	109.93 ± 91.79
G3: IMP301 + MB + LENS	1.61 ± 0.34	2.19 ± 0.73	0.92 ± 0.01	0.86 ± 0.02	179.22 ± 44.34
G4: DOXIL	1.89 ± 0.39	2.52 ± 0.49	0.95 ± 0.05	0.86 ± 0.04	99.82 ± 24.58
G5: IMP301 w/o DOXIL	1.92 ± 0.49	3.56 ± 1.19	0.99 ± 0.03	0.97 ± 0.04	0.00 ± 0.00

### Biodistribution of IMP301 and DOX after LENS-applied treatment

**Figure 5:**
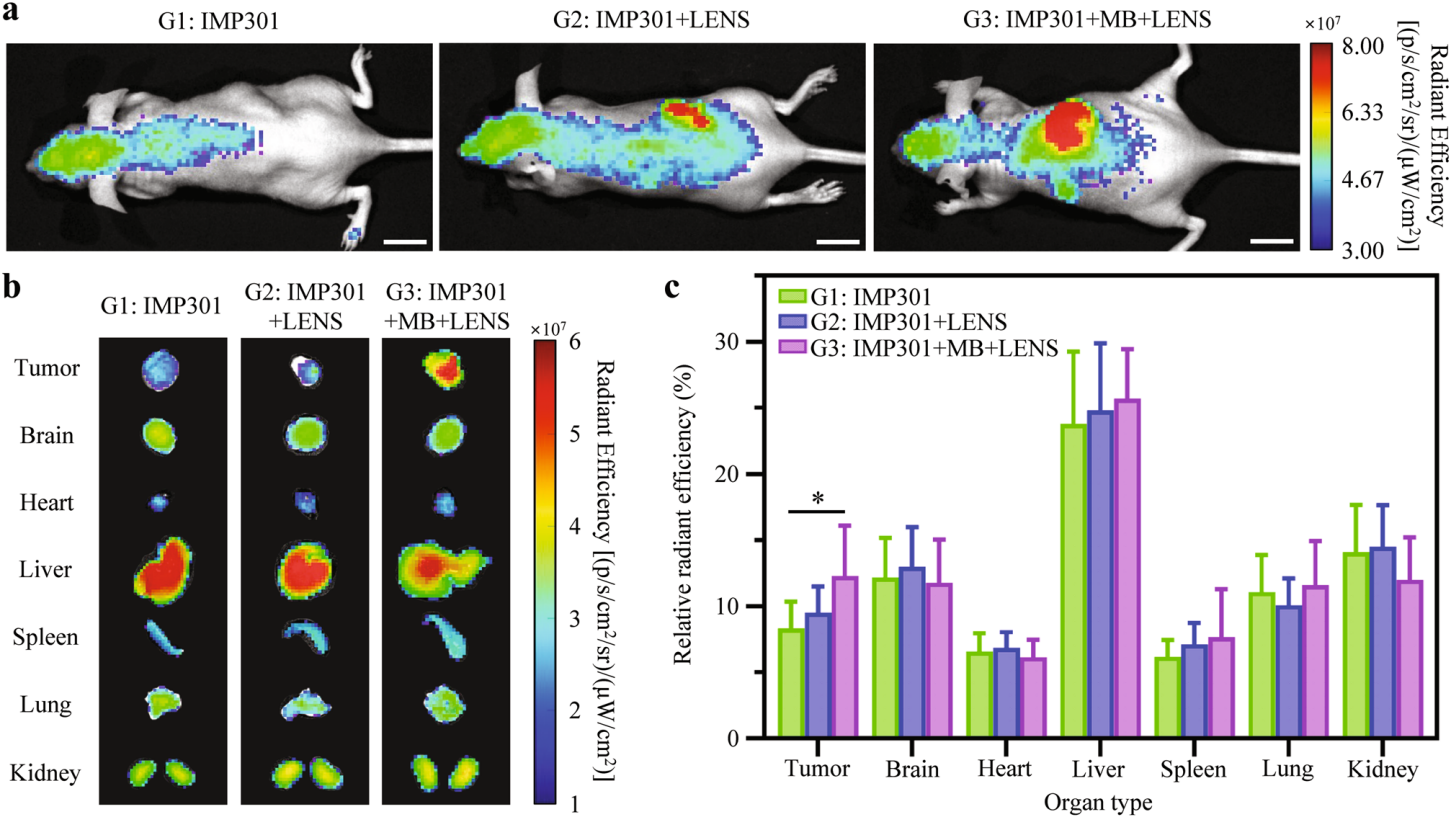
Biodistribution of IMP301 with LENS-Enhanced Treatment in Murine Model. (**a**) Representative qualitative in vivo body biodistribution profiles of IMP301 with each treatment (G1: IMP301, G2: IMP301 + LENS-applied treatment, and G3: IMP301 + MB + LENS-applied treatment) by fluorescence imaging. DOXIL group (G4) is excluded from the bioluminescence analysis due to the unoptimized imaging condition for DOXIL visualization. Additionally, IMP301 without the DOXIL group (G5) is excluded from the analysis since our analysis focused on assessing drug biodistribution in combination with IMP301 under US exposure. This group also remained transparent due to the absence of DOX. The scale bar indicates 1 cm. (**b**) Ex vivo fluorescence imaging of dissected organ biodistribution of antitumor drug with each treatment (G1, G2, and G3) to validate the sonosensitive effect of IMP301 by LENS. (**c**) Relative radiant efficiency of tumor, brain, heart, liver, spleen, lung, and kidney in the corresponding organ biodistribution images after the treatments (Tumor, G1 vs. G3: *p < 0.05).

As shown in Fig. 5a, the IMP301 signal accumulated in the whole murine body and was primarily distributed in the brain and liver in the non-LENS-treated group (G1). However, IMP301 signals were mainly located at the tumor sites in the LENS-treated groups (G2 and G3). These data demonstrated that the LENS-applied treatment could actively improve IMP301 accumulation at the tumor sites. The IVIS imaging conditions were optimized to ensure precise quantification of IMP301 in treatments involving IMP301 (G1, G2, and G3). However, the DOXIL group (G4) shows limited DOX accumulation in the tumor region due to the imaging conditions not being optimized for DOXIL visualization. The fluorescent signal measurement via IVIS was not conducted for the IMP301 without the DOXIL group (G5) as this aspect of the analysis was specifically designed to evaluate the biodistribution of the drug in conjunction with because the IVIS analysis was to assess the biodistribution of the drug when combined with IMP301 under US exposure. The IMP301 solution without DOXIL (G5) remained transparent due to the absence of DOX. To validate the sonosensitive effect of IMP301 by LENS on organ-dependent targeting efficacy, we selected three groups—G1, G2, and G3. After each treatment process, we harvested major organs and tumors to conduct ex vivo bioluminescence imaging for further evaluation of organ-dependent targeting efficacy (Fig. 5b). Compared with the LENS-treated group, IMP301 was found to accumulate mainly in the liver and kidney without LENS-treatment. In particular, the combination treatment with IMP301 + MB + LENS-applied treatment (G3) achieved better tumor-targeting ability than IMP301 + LENS-applied treatment (G2). When quantifying the corresponding bioluminescence intensities, the IMP301 signals at the tumor sites for the IMP301 + MB + LENS-treated group (G3) were significantly higher than that in the non-LENS-treated group (G1) (Tumor, G1 vs. G3: *p < 0.05) (Fig. 5c).

**Figure 6:**
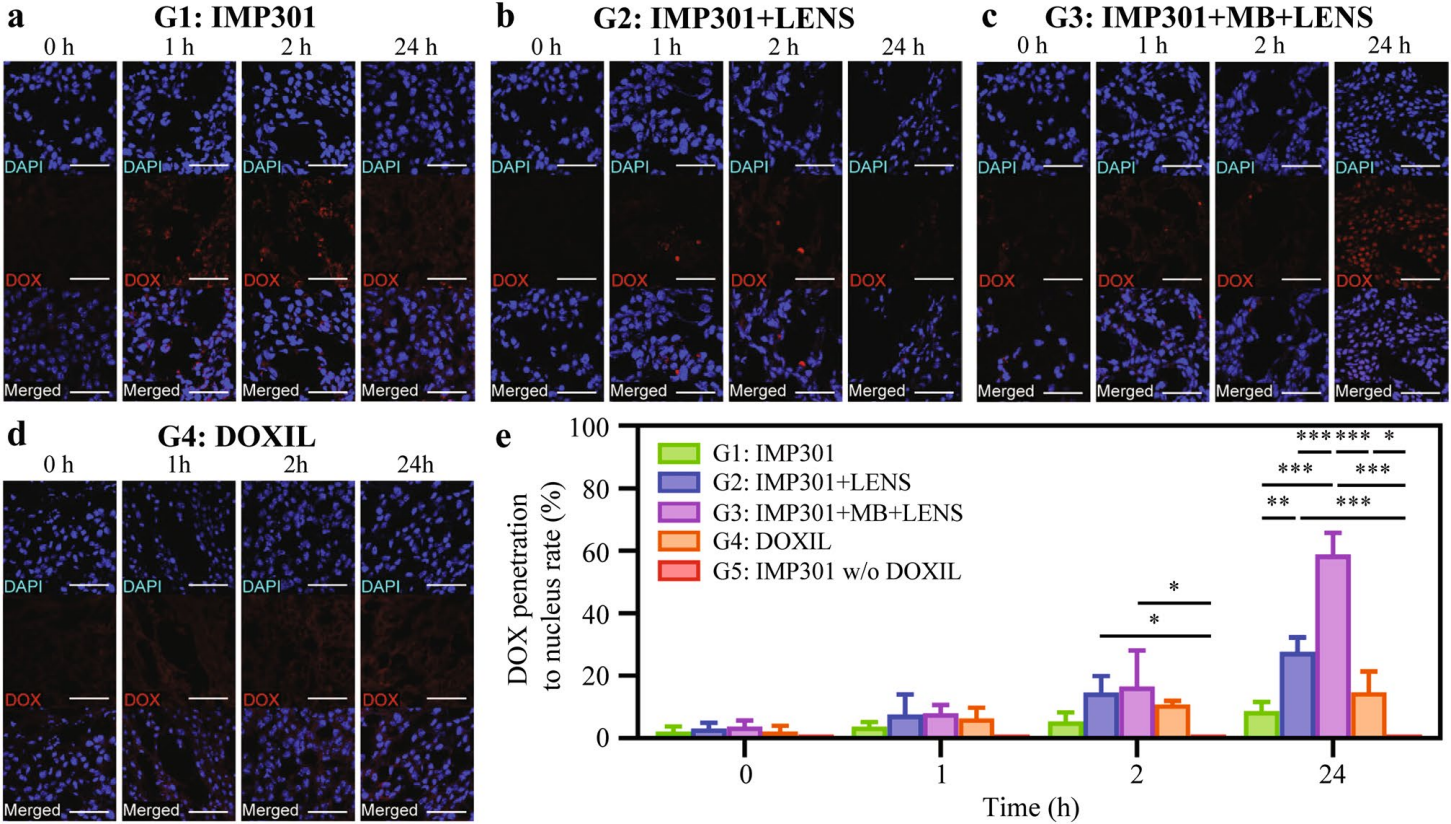
Antitumor Uptake in Subcutaneous Tumors after LENS-Enhanced Treatment: In vitro Confocal Laser Scanning Microscopy (CLSM) Imaging. Representative CLSM images of DAPI- (blue) and DOX-labeled (red) antitumor drug uptake in the subcutaneous tumor after each treatment (**a**) G1: IMP301, (**b**) G2: IMP301 + LENS, (**c**) G3: IMP301 + MB + LENS and (**d**) G4: DOXIL-only for elevated time (1, 2, and 24 h). IMP301 without DOXIL group (G5) does not exhibit any fluorescence signals of DOX on its CLSM image due to the absence of DOX. The scale bar indicates 50 μm. (**e**) Antitumor drug (DOX) penetration to nucleus rate after each treatment (G1-G5) for elevated time (0, 1, 2, and 24 h) (2 h, G2 vs. G5: *p < 0.05; 2 h, G3 vs. G5: *p < 0.05; 24 h, G1 vs. G2: **p < 0.001; 24 h, G1 vs. G3: ***p < 0.0001; 24 h, G2 vs. G3: ***p < 0.0001; 24 h, G2 vs. G5: ***p < 0.0001; 24 h, G3 vs. G4: ***p < 0.0001; 24 h, G3 vs. G5: ***p < 0.0001; 24 h, G4 vs. G5: *p < 0.05).

The penetration rate of antitumor drugs into the nucleus is critical in evaluating their efficacy. As shown in Fig. 6a–d, treatment groups with IMP301 (G1, G2, and G3) showed similar intratumoral DOX delivery as the DOXIL-only treatment group (G4), demonstrating drug into tumor nuclei (each treatment after an hour). However, IMP301 with LENS-applied groups (G2 and G3) exhibited deeper penetration and higher cellular uptake with higher DOX signal intensity than other treatment groups due to a better merged DOX signal with cell nuclei fluorescence (each treatment after 2 and 24 h). For G5, IMP301 without DOXIL as a treatment material precluded the confirmation of DOX signals after treatment. Here, we calculated this rate by determining the area of overlap between DOX-labeled and DAPI-labeled regions within the ROI of the confocal laser scanning microscopy (CLSM) image and dividing it by the DAPI-labeled region in the same ROI (Fig. 6e). Our graph analysis revealed that the DOX penetration rate into the nucleus increased over time in all treatment groups except for G5. Notably, the rate of increase was highest in G3, followed by G2, while G4 and G1 exhibited similar rates of increase (DOX penetration rate at *t* = 1 h, G1: 3.64 ± 1.54%, G2: 7.61 ± 6.33%, G3: 7.90 ± 2.64%, G4: 6.30 ± 3.49%, G5: 0%; DOX penetration rate at *t* = 2 h, G1: 5.49 ± 2.82%, G2: 14.60 ± 5.32%, G3: 16.50 ± 11.59%, G4: 10.82 ± 1.09%, G5: 0%; DOX penetration rate at *t* = 24 h, G1: 8.68 ± 2.91%, G2: 27.62 ± 4.61%, G3: 58.78 ± 6.95%, G4: 14.67 ± 6.73%, G5: 0%). We observed a significant difference in the DOX penetration rate between the G5 group and both the G2 and G3 groups at *t* = 2 h (G2 vs. G5: *p < 0.05; G3 vs. G5: *p < 0.05). Furthermore, significant differences were observed between each group, except for comparison between G1 and G4, as well as G1 and G5, at *t* = 24 h (G1 vs. G2: **p < 0.001; G1 vs. G3: ***p < 0.0001; G2 vs. G3: ***p < 0.0001; G2 vs. G5: ***p < 0.0001; G3 vs. G4: ***p < 0.0001; G3 vs. G5: ***p < 0.0001; G4 vs. G5: *p < 0.05). It is worth noting the DOX signal intensity within tumor tissue nuclei in G3 was higher than those in the other groups at *t* = 2 h and 24 h. These results highlight the significant group variations enabling distinct treatment responses and outcomes.

## Discussion

The study found that the combination of US-responsive liposomal DOX and FUS exposure enhanced by LENS application efficiently delivered antitumor drugs into melanoma. This approach results in their qualitative therapeutic effects (G2 and G3) compared with IMP301 alone (G1) and DOXIL (G4), which does not reveal any significant differences in the tumor volume and body weight with IMP301 without DOXIL (G5). When comparing to the G5 group (IMP301 without DOXIL), we found significant differences in the relative tumor volume for both the G2 (IMP301 + LENS) and G3 (IMP301 + MB + LENS) groups. Upon examining the intratumoral drug concentration results, we observed a significant difference exclusively in the G3 group compared to the results obtained from the G4 and G5 groups. A comparison of the relative radiant efficiency in tumors between the G1 and G3 groups revealed a substantially higher tumor accumulation ratio in the G3 group. Additionally, in the CLSM imaging analysis, we confirmed a significantly higher rate of DOX penetration to the tumor nucleus over time in both the G2 and G3 groups compared to the other treatment groups. These findings highlight the significant feasibility of our proposed drug delivery system in cancer therapy, showing the enhanced tumor-targeting capabilities and the potential for therapeutic effects. The enhanced drug delivery strategy involves the administration of liposomal chemotherapeutic agents with MBs and then exposing the tumor tissues under US pressure.

There are two main advantages of inducing drug release using US and MB. It enables tumor characterization utilizing the CEUS system, providing valuable insights into tumor characteristics and morphology. Another notable aspect of our study is a target-specific drug delivery approach achieved by modulating the power and shape of the US beam. This capability can allow for precise and controlled drug delivery to the intended target, further enhancing the efficacy and accuracy of the treatment.

CEUS enables tumor imaging by visualizing intratumoral blood perfusion where the MBs flow. Following LENS-applied treatment, the treated tumor sites exhibit a more significant and continuous reduction in normalized US intensity than untreated sites. This is because FUS exposure by LENS-applied treatment can promote MB cavitation, leading to MB destruction^12–15^. These results indicated that LENS-applied CEUS imaging enabled tumor imaging and real-time treatment monitoring simultaneously.

MB oscillation by cavitation leads to microstreaming and acoustic pressure, generating high shear stress on the liposomal carrier and cell membrane. Encapsulated drugs in IMP301 can be released by FUS exposure alone, but FUS-mediated MB cavitation enhances drug release and intratumoral drug penetration^14,16^. The mechanical stress promotes the disruption of the phospholipid shell of IMP301 and improves the dispersive transport and diffusivity of DOX to tumor cells^17,18^. The combination of LENS-induced MB cavitation and liposomal drug carriers can improve therapeutic efficacy by providing high vascular permeability, increased interstitial transport, enhanced cellular uptake, hydrostatic pressure reduction, and loosened intercellular junctions in terms of biophysics^19–22^. This study confirms the feasibility of our proposed approach through observations of the tumor growth rate over time and the increased intratumoral DOX concentration by the IMP301 + MB + LENS-applied treatment (G3). However, weight loss might be regarded as an adverse effect of DOX as an antitumor drug. Without FUS, drug-loaded liposomes are hindered by the high interstitial fluid pressure^23–25^. Moreover, liposomal formulations enable intratumoral concentration due to their characteristics, including longer half-life and slower clearance^26,27^.

DOX accumulated mainly in the liver and kidney without the LENS, compared with the LENS-applied group. It is consistent with previous studies due to the hepatic metabolic conversion and renal clearance^28,29^. In particular, the combination treatment with IMP301 + MB + LENS-applied treatment (G3) achieved better tumor-targeting ability than IMP301 + LENS-applied treatment (G2) due to the FUS-mediated MBs cavitation effect. In contrast to the tumor-targeting efficacy of the combination of IMP301, MBs, and LENS-applied treatment as shown in Fig. 6, CLSM images of the major organs of each treated group (G1, G2, and G3) led to no significant increase in DOX signal intensity over time after the treatments (Supplementary Figs. S1–S3). These results also indicate that FUS exposure, combined with MBs, can enhance the permeability of the intratumoral vasculature and tumor tissues, facilitating target-specific drug delivery comparable to previous studies^30,31^.

This study is subject to certain limitations. Specifically, when performing statistical analyses to evaluate the therapeutic effects of each treatment, we did not observe statistically significant differences among them directly in the results of relative tumor volume and body weight. We attribute this limitation to the highly aggressive growth rate exhibited by the melanoma tumor used as the target in our study. Consequently, the tumor size in the 2nd and 3rd treatment stages was considerably more extensive compared to the area of the treated region. In future investigations, we will closely examine significant differences in the tumor progression delay effect on the model exhibiting less aggressive growth rates. It will result when the treated tumor size remains relatively small, enabling the comprehensive targeting of the entire tumor region during the treatment process. Secondly, the bioluminescence imaging conditions used in this study for IVIS analysis posed challenges when attempting to compare all treatment methods quantitatively. Usually, DOX exhibits bioluminescence when excitation/emission wavelengths (Ex/Em) are 488/530 nm. However, in this study, we employed a near-infrared wavelength range with Ex/Em of 640/710 nm to detect IMP301. This imaging condition was chosen to reduce autofluorescence signals in vivo but simultaneously resulted in relatively low absorbance. Furthermore, since DOXIL is a passively targeted liposomal drug, achieving a precise observation of tumor accumulation may be more challenging than treatments based on IMP301. Therefore, to accurately assess the biodistribution of each treatment using the IVIS analysis, optimized imaging conditions for both DOXIL and IMP301 are necessary. These limitations warrant further exploration and consideration in future studies. Third limitations include a lack of in-depth discussion on pathological changes and adverse effects on surrounding tissues. Although we examined the efficacy of our therapeutic approach using micro-CT imaging and bioluminescence imaging, it would be necessary to elucidate the therapeutic effects of our proposed treatment on tumor cells and nearby healthy cells with histopathologic analysis and deal with their in-depth discussion, compared to previous studies^14,30^. Furthermore, large animal models should consider unintentional acoustic reflection and scattering. With future animal and tumor model studies, we will demonstrate the capability of US beam control using a LENS system based on acoustic lens geometry. Pharmacodynamic analysis of IMP301 is also needed to understand drug metabolism and tumor metastasis. Future clinical studies will confirm the treatment's combined therapeutic effects on various tumors.

We propose combining US-responsive liposomal drug carriers, MBs, and LENS-applied treatment for effective tumor progression delay and monitoring. FUS exposure by LENS leads to on-demand drug release from liposomal carriers and improved tumor penetration and cellular uptake, demonstrating the potential for tumor progression delay effect. Our approach showed the feasibility and potential for the tumor progression delay effect in a murine melanoma model, offering a promising strategy for precise tumor treatment and drug release control, and could extend to potential benefits for tumor diagnosis. Considering the perspective of an in vivo feasibility study introducing the synergistic combination treatment, these findings hold significant importance due to expansion into diverse treatment modalities employing various acoustic lens conditions.

## Methods

### LENS system with sonosensitive liposome for drug delivery

A 3D-printed polylactic acid mold was created for a convex acoustic lens using acoustic modeling software (COMSOL Multiphysics 5.3a). The lens was then replicated using polydimethylsiloxane and attached to a 64-element phased-array transducer (3Sp-D, Humanscan Co. Ltd.) with a center frequency of 3.3 MHz. This configuration enabled the creation of FUS beams, where the US waves converged to facilitate localized US exposure treatment specifically for US-induced drug delivery. The custom US pulser system, controlled by a field-programmable gate array (FPGA) device (Spartan-6 FPGA, Xilinx), enabled simultaneous control of pulse repetition frequency (PRF), duty cycle, and acoustic power. FUS exposure was performed at 2.34 MPa acoustic pressure, 100 kHz PRF, and a 9% duty cycle, which was optimized by an MB destruction experiment setup and analysis method in our previous study^13^. The acoustic lens-attached US system allows for precise control of the treated region based on the size and location of the tumor tissue. It can manipulate the US exposure area (both its size and depth) by modifying the geometry of the acoustic lens attached to the US transducer^13^.

The newly developed LENS system enhanced target-specific drug delivery and selective release of US-sensitive liposomes encapsulating DOX (IMP301; IMGT Co. Ltd, Seongnam, Korea) chemotherapeutics dependent on MB cavitation under FUS exposure (Fig. 1). The US-sensitive liposome encapsulating DOX, IMP301, was designed for DOX release under specific conditions of FUS pressure based on the composition and ratio of phospholipids. Previous studies have described the fabrication process, physicochemical characteristics, and sonosensitivity of IMP301^14^. Briefly, the complex lipid composition of IMP301 was fabricated into multilamellar vesicles, DOX was encapsulated into the intraliposomal dispersion, and the final DOX concentration of IMP301 was 2 mg/mL and stored at 2–8 °C. For a quantitative comparison of therapeutic effects across the treatments, we ensured that the final DOX concentration of IMP301 corresponded to the DOX concentration (2 mg/mL) of the DOXIL product (CAELYX™) employed in this study. In the fabrication process of multilamellar vesicles using complex lipids, we utilized the sonoresponsive liposome without DOX loaded to create the comparison group for IMP301, distinct from DOXIL.

### Experimental design for drug delivery activated by LENS

This study was approved by the Institutional Animal Care and Use Committee (IACUC; No. 20-0101-S1A0) and was performed under the Guide for the IACUC and the National Institute of Health Guide for the Care and Use of Laboratory Animals. All experiments were performed according to relevant regulations and the ARRIVE guidelines.

**Figure 7:**
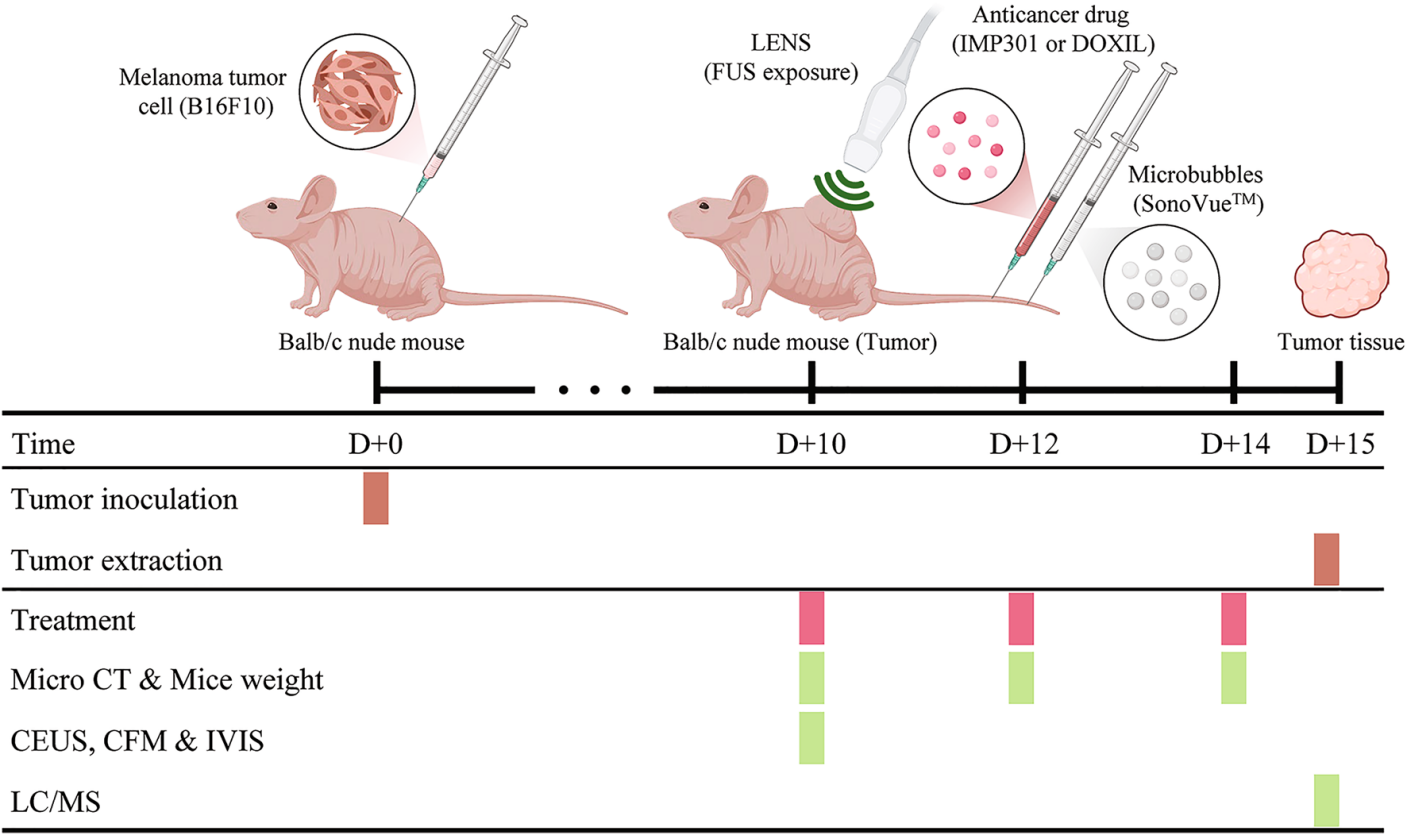
Experimental Timeline for Targeted Drug Delivery using LENS in Murine Melanoma Model. Timeline of the experimental procedure with murine subcutaneous melanoma model to verify target-specific drug delivery treatment efficacy and toxicity of IMP301 with LENS-applied treatment. Each treatment following antitumor drug administration and the subsequent drug delivery evaluation by micro-CT, mice weight, CEUS, CFM, IVIS, and LC/MS.

B16-F10 melanoma cells were obtained from the Korea Cell Line Bank and cultured in Dulbecco's modified Eagle's medium (Welgene, Gyeongsan, Korea) containing 10% fetal bovine serum (Welgene, Gyeongsan, Korea) and 1% fetal bovine serum. A murine melanoma model was created by injecting melanoma cells (2 × 10^5^/100 μL) subcutaneously into the backs of immunodeficient mice (Balb/c, Orient Bio., Seongnam, Korea). For tumor implantation and imaging examinations, mice were sedated with an intraperitoneal injection of a mixture of zolazepam (Zoletil; Virbac, Carros, France) 5 mg/kg and xylazine hydrochloride (Rompun 2%; Bayer Korea, Seoul, Korea). Ten days after tumor injection, tumor-bearing mice were selected for at least a tumor volume of 200 mm^3^ and then treated according to each treatment strategy on 10, 12, and 14 days from the day of tumor injection (i.e., three treatments in total) (Fig. 7). For all mice in the LENS-treated groups, each tumor was immediately exposed to LENS for 5 min after injection of the treatment material. The G3 group with MB injection was treated with IMP301 injection and immediately exposed to LENS for 5 min following MB injection (0.3 mL through the tail vein). On the 15th day after the tumor injection, all mice were euthanized with a lethal dose of sodium pentobarbital (200 mg/kg body weight, intraperitoneal), and the tumors were carefully removed.

### Contrast-Enhanced ultrasound imaging to LENS-applied effect

A CEUS examination was performed on the first treated mice to optimize the LENS application time, affecting the MB cavitation effect. All CEUS examinations were performed by an experienced radiologist (with 14 years of clinical US examination experience) using a Mindray DC-80 US machine (Mindray, Shenzhen, China) with a linear transducer (L12-3E, center frequency of 7 MHz, mechanical index of 0.134, dynamic range 1 of 21 dB, gain of 61%). The MB (SonoVue®, Bracco, Milan, Italy) US contrast agent was injected through the tail vein of each mouse with a 31-gauge needle with a bolus injection of 0.3 mL following 0.1 mL of saline flush. After the MB injection, continuous CEUS data were obtained for 15 min.

The echo signal variations were quantified using the normalized US intensity values in the ROI of the untreated tumor, treated tumor, and background on the CEUS images. Each normalized US intensity was calculated as follows:

1 $$I_{{{\text{norm}}}} = I_{{{\text{ROI}}}} /I_{\max }$$

where *I*_norm_ is the normalized US intensity value, *I*_ROI_ is the averaged US intensity of the ROI (1.1 mm × 1.1 mm), and *I*_max_ is the maximum US intensity in the CEUS image.

### Therapeutic effect by LENS-applied treatment

Each tumor volume measurement using micro-CT was performed on the day of tumor treatment. The CT imaging parameters were a peak voltage of 90 kVp, current of 88 µA, standard resolution of 120 µm, nominal resolution, and 36 mm field-of-view for one-bed acquisition. The mice were intravenously injected through the tail vein with 0.3 mL of the iodine contrast agent iohexol-350 (Bonorex 350; Central Medical Service, Seoul, Korea; 350 mg iodine per mL) to obtain a contrast-enhanced CT image. All tumor volumes were acquired by three-dimensional measurement of axial and coronal axes with contrast-enhanced micro-CT images. Growth rates were calculated by dividing the current volume by the previous volume of tumors.

The concentration of antitumor drug in the tumor collected an hour after the final step of each treatment was analyzed using liquid chromatography–mass spectroscopy (LC/MS). Intratumoral DOX concentrations were assessed using a Triple Quadrupole 6500 + System (AB Sciex, Framingham, MA, USA), which consisted of a SHIMADZU Nexera (Shimadzu Corporation, Kyoto, Japan) ultrahigh-performance liquid chromatograph coupled with a hybrid triple quadrupole/linear ion trap mass spectrometer. Extracted tumors were homogenized with acid alcohol in phosphate buffer, as previously described^12,13,32^. The DOX concentration in each sample was further normalized using the weight of the corresponding tumor and was measured between 10 and 250 ng/mL.

### Biodistribution of DOX after LENS-applied Treatment

The biodistribution of the anticancer drug from each treatment was investigated using an in vivo bioluminescence imaging system to examine their tumor-targeting performance. Bioluminescence imaging was performed on the day of sacrifice using an IVIS® Lumina II imaging system (Xenogen Corp., Alameda, CA, USA). The anesthetized mice in the ventral position were placed in the chamber of the IVIS® Lumina II imaging system 24 h after the last treatment. A filter set (excitation: 640 nm; emission: 710 nm) was used to acquire the signal for IMP301 in vivo while simultaneously minimizing autofluorescence signals^14^. Under the specific IVIS imaging parameters, the variations in radiant efficiency with varying concentrations of IMP301 can be quantitatively observed (Supplementary Figure S4). ROI was drawn on the tumors to assess the emitted DOX signal. The mice were sacrificed, the liver, lung, spleen, kidney, heart, and tumors were extracted, and ex vivo bioluminescence images were obtained to quantify the signals in each organ. The results were expressed as the average radiant efficiency in units of photons/second within the ROI ([p/s/cm^2^/sr]/[μW/cm^2^]) using Living Image 2.5 software (Caliper, Alameda, CA, USA). The relative radiant efficiency for each tumor or organ, which means the normalized average radiant efficiency of each organ by the cumulative average radiant efficiencies of the total tumor and organs, was calculated below,

2 $$\begin{aligned} & {\text{Relative}}\;{\text{radiant}}\;{\text{efficiency }} \\ & = \, \frac{{{\text{Average}}\;{\text{radiant}}\;{\text{efficiency}}\;{\text{of}}\;{\text{each}}\;{\text{tumor}}\;{\text{or}}\;{\text{organ}}}}{{{\text{Cumulative}}\;{\text{average}}\;{\text{radiant}}\;{\text{efficiencies}}\;{\text{from}}\;{\text{the}}\,{\text{total}}\;{\text{tumor}}\;{\text{and}}\;{\text{organs}}}} \times 100 \, (\% ) \\ \end{aligned}$$

This value evaluates the relative accumulation of drugs or nanocarriers within each tumor and organ with treatments with IMP301 and LENS. The DOX signal was detected to visualize the in vivo tumor penetration of the antitumor drug IMP301 using confocal microscopy. DOX signals in the tumor and main organs were observed by confocal scanning microscopy [LEICA TCS SP8 with an inverted microscope (DMI 600 B, Leica Biosystems, Buffalo Grove, IL, USA)]. Fluorescent 4′,6-diamidino-2-phenylindole (DAPI) staining was performed, and DOX fluorescence was detected at excitation and emission wavelengths of 488 and 530 nm, respectively. Post-imaging data were processed using the Aperio ImageScope software (Version 12.3, Leica Biosystems, Wetzlar, Germany) for randomly selected regions of each tumor. The antitumor drug penetration to nucleus rate is calculated by dividing the area of overlap between the DOX-labeled region and the DAPI-labeled region in the ROI of the CLSM image by the DAPI-labeled region in the same ROI, as below,

3 $${\text{DOX}}\;{\text{penetration}}\;{\text{to}}\;{\text{nucleus}}\;{\text{rate}} = \frac{{{\text{DOX-labeled}}\;{\text{region}} \cap {\text{DAPI-labeled}}\;{\text{region}}}}{{{\text{DAPI-labeled}}\;{\text{region}}}} \times 100 \, (\% )$$

### Statistical analysis

All statistical analyses were performed using GraphPad Prism 8.0.1 (GraphPad Software Inc., San Diego, CA, USA). One-way analysis of variance (ANOVA) was performed to analyze the difference in mean of the three groups (CEUS analysis). Inter-group analyses were performed using two-way ANOVA for multiple comparisons. All results are represented as mean ± standard deviation (SD), and a *p*-value of < 0.05 was considered statistically significant between the corresponding parameters.

### Supplementary Information

Supplementary Figures.

## Data Availability

The datasets generated and/or analyzed during the current study are available from the corresponding author upon reasonable request.
