# Supplementary material for: Drug delivery by sonosensitive liposome and microbubble with acoustic-lens attached ultrasound: an in vivo feasibility study in a murine melanoma model
Source: Sci Rep. 2023 Sep 22;13:15798. doi: 10.1038/s41598-023-42786-8 (PMC10517155; doi:10.1038/s41598-023-42786-8)
Supplement: Supplementary file 1 — Supplementary Figures. [file 41598_2023_42786_MOESM1_ESM.docx]

**Table of Content**

Supplementary Figure S1–S4


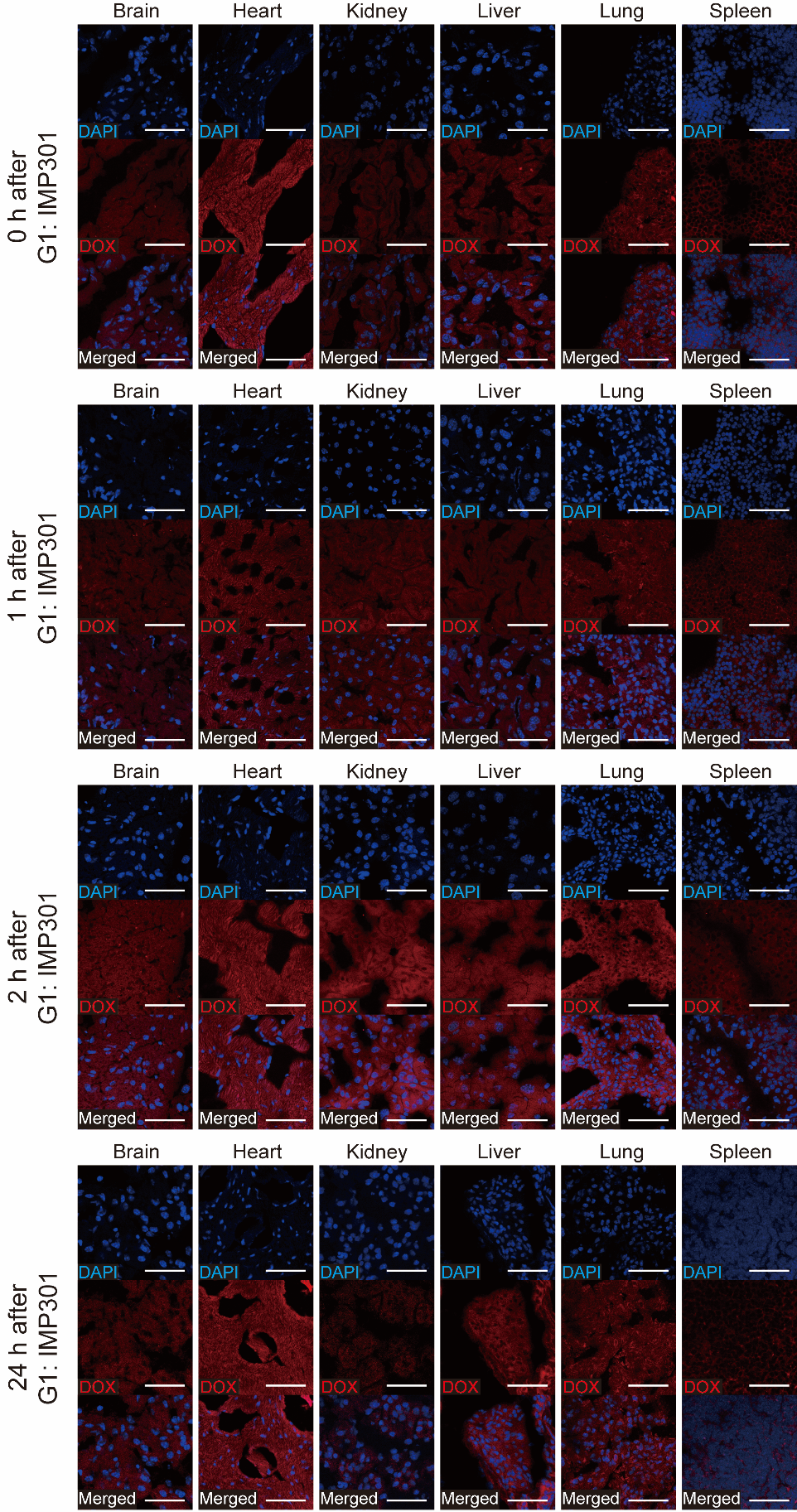


**Supplementary Figure S1. In vivo bio-distribution of drug from IMP301 treated group (G1).** Quantification of fluorescence intensity of major organs at 0 h, 1 h, 2 h and 24 h after the IMP301+MB treatment.


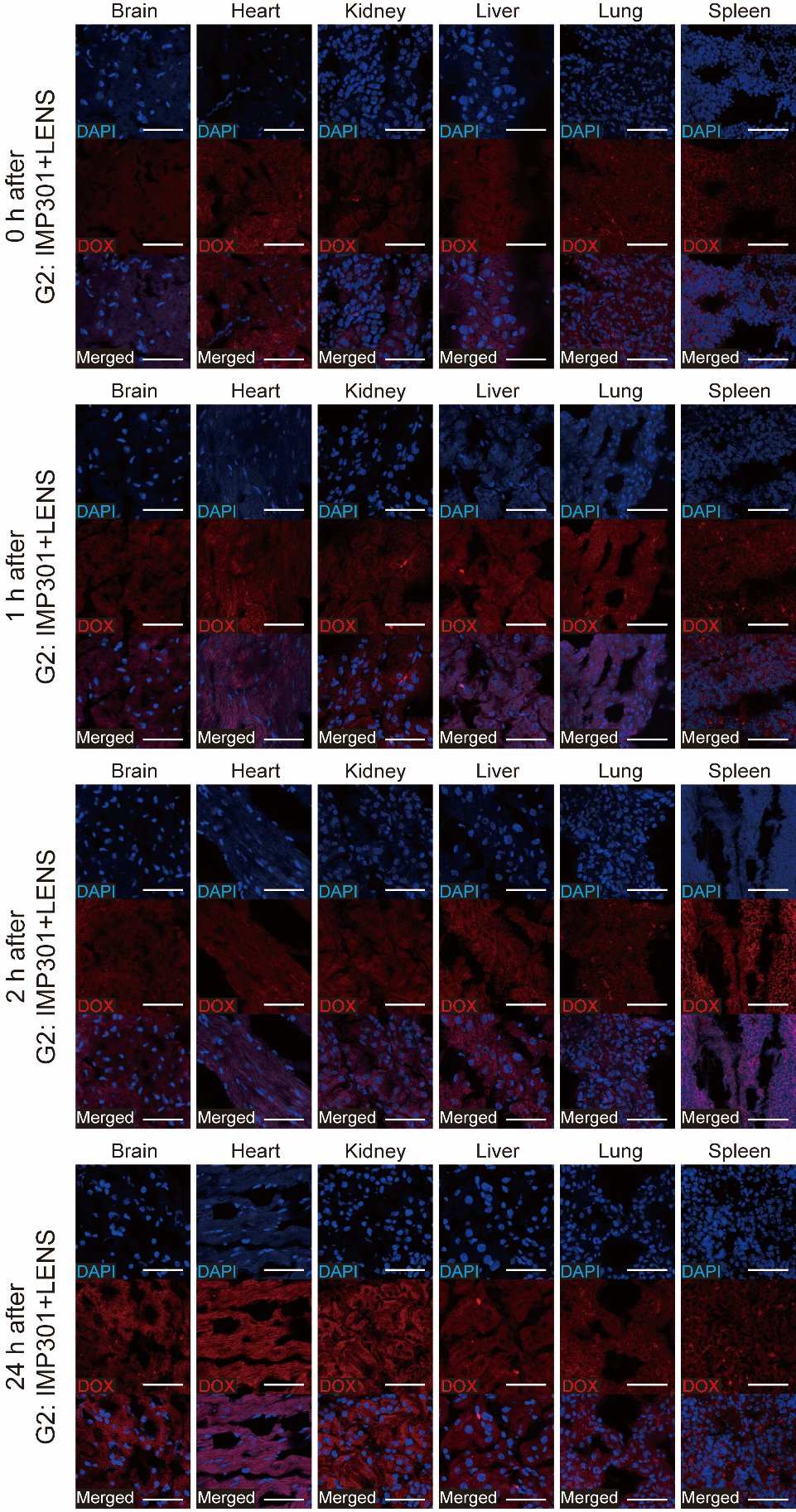


**Supplementary Figure S2. In vivo bio-distribution of drug from IMP301+LENS treated group (G2)**. Quantification of fluorescence intensity of major organs at 0 h, 1 h, 2 h and 24 h after the IMP301+LENS treatment.


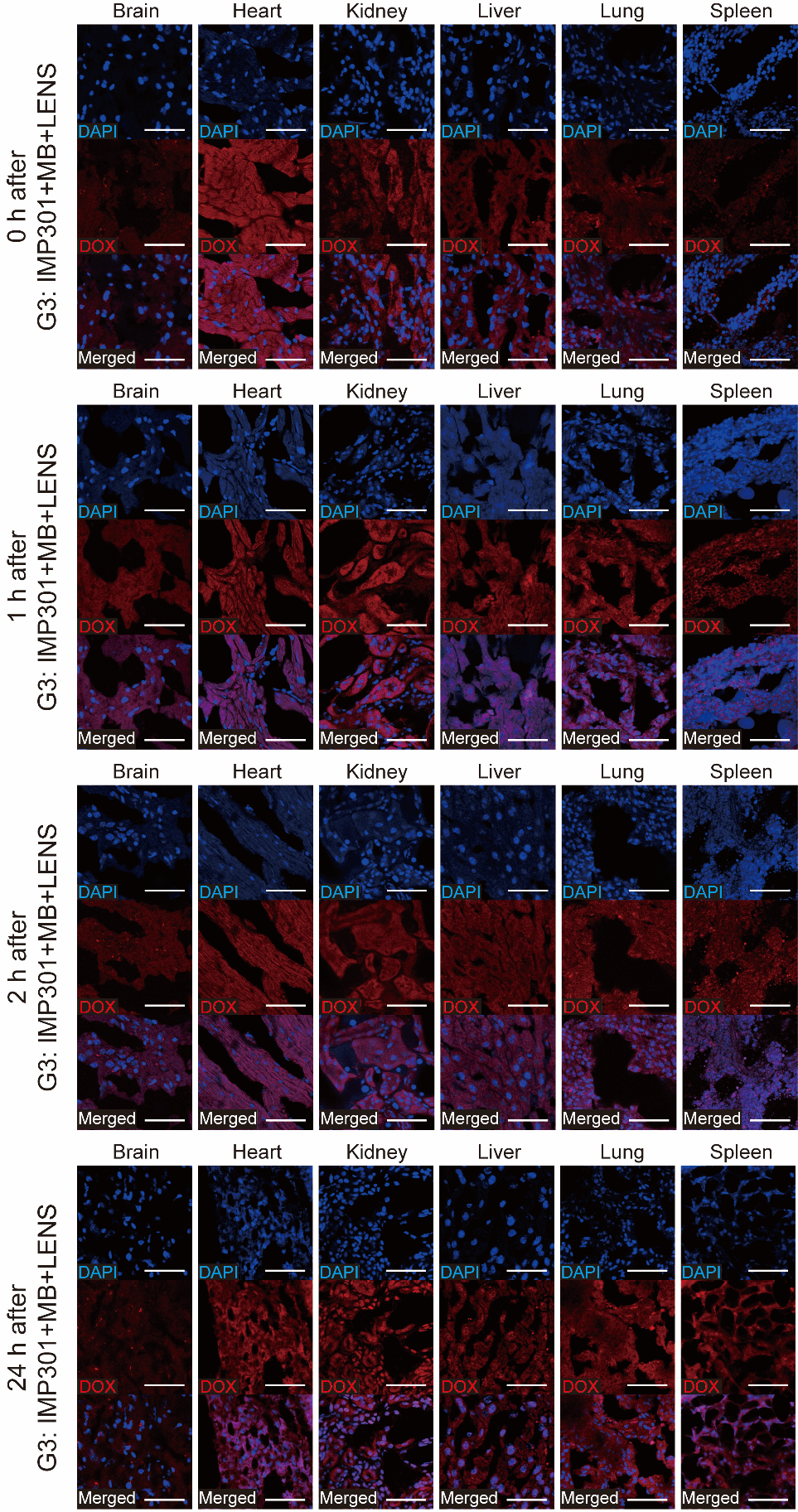


**Supplementary Figure S3. In vivo bio-distribution of drug from IMP301+MB+LENS treated group (G3).** Quantification of fluorescence intensity of major organs at 0 h, 1 h, 2 h and 24 h after the IMP301+MB+LENS treatment.


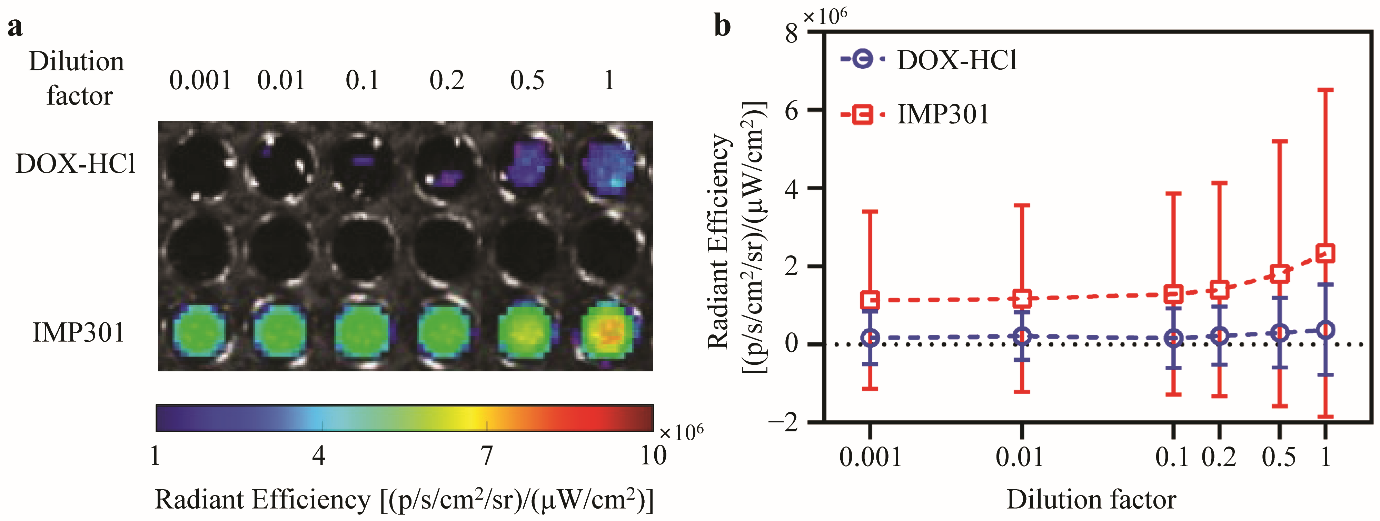


**Supplementary Figure S4. In vitro calibration of excitation and emission wavelengths condition for optimal in vivo IVIS analysis.** (a) In vitro fluorescence imaging of DOX-HCl and IMP301 solutions to calibrate fluorescence intensity with varying their concentrations within well plates under 640/710 (Ex/Em) condition. The solutions were diluted with saline, and a dilution factor of 1 corresponds to DOX concentration of IMP301 of 2 mg/ml and DOX concentration of 2 mg/ml. (b) Radiant efficiency assessment of DOX-HCl and IMP301 solutions with varying dilution factors.
